# Does the combining effects of energy and consideration of financial development lead to environmental burden: social perspective of energy finance?

**DOI:** 10.1007/s11356-021-13423-6

**Published:** 2021-03-27

**Authors:** Fengsheng Chien, Ka Yin Chau, Sri Utami Ady, YunQian Zhang, Quyen Ha Tran, Talla M. Aldeehani

**Affiliations:** 1 School of Finance and Accounting, Fuzhou University of International Studies and Trade, Fuzhou, Fujian China; 2 Faculty of Business, City University of Macau, Macau, China; 3 Economic and Business Faculty, University of Dr. Soetomo, Surabaya, Indonesia; 4 Faculty of International Tourism and Management, City University of Macau, Macau, China; 5 University of Economics Ho Chi Minh City, Ho Chi Minh City, Vietnam; 6 College of Business Administration, Kuwait University, Kuwait City, Kuwait

**Keywords:** Economics of energy, Energy finance, Renewable energy, Data envelopment analysis, G7

## Abstract

In light of the rapidly growing industrialization in BRICS and G7 regions, thorough energy, financials, and environmental analyses are essential for sustainable financial development in these countries. In this context, this work analyzes the relationship between energy, financial, and environmental sustainability and the regions’ social performance. Data from 2000 to 2017 is analyzed through a data envelopment analysis (DEA) like a composite index. Results show China and Brazil’s better performance in the region, with a sustainability score of 0.96, India was the third, followed by South Africa and Russia. Japan, the UK, and the USA were the most energy-efficient countries for five consecutive years. A 0.18%, 0.27%, 0.22%, 0.09%, 0.31%, and 0.32% reduction in carbon emission is observed with a 1% increase in R&D costs by Canada, France, Germany, Italy, Japan, and the USA, respectively. This work contributes to the existing literature regarding an eco-friendly sustainable policy design for the G7 countries based on multiple indicators.

## Introduction

Climate change poses a severe threat to future generations and must be tackled through practical solutions. Policymakers and scientists propose effective, sustainable development solutions to reduce the adverse effects of climate change. Directly dependent on the challenges in the transformation of energy systems, sustainable development should be considered a priority in policy design as it improves people’s living standards without negatively affecting the environment. The development agenda “Transforming our world: the United Nations General Assembly adopted the 2030 Agenda for Sustainable Development” (Mohsin et al. 2019a) on November 25, 2015. The 2030 Agenda is to create a strategic partnership at all levels through the idea of “leaving no one behind” (Yang et al. 2021; He et al. 2020, and Mohsin et al. 2020b). The Sustainable Development Goals (SDGs) were developed, on January 1, 2016, to enable sustainable development in various areas, such as social, environmental, and financial. The efficiency of various financial sectors can be increased with a simultaneous decrease in environmental pollution by tackling climate change (Si et al. 2020; Mier and Weissbart 2020). China’s impressive financial development shows how the new sectors and effective financial development affect GHG emissions (Anser et al. 2018; Anser 2019, and Anser et al. 2020g). It is essential to measure different economies’ environmental performance due to GHG emissions’ debilitating effect on the environment. With a continuous effort to limit the rise in temperature to 1.5°C, such initiatives help formulate a precise summary for growth evaluation and form environmental objectives to keep the average temperature rise below 2°C (Mohsin et al. 2018a; Mohsin et al. 2019c).

In recent years, countries worldwide have liberalized their markets to enhance their economic efficiency, resulting in intensified competitiveness among companies. As a consequence, companies have raised their research and development (R&D) budgets. Domestic markets would benefit immensely from these changes, as domestic products have been more modernized, drawing domestic consumers (Liu et al. 2020a). Consequently, demand for imports has declined in countries with a high level of creativity and R&D investment. R&D investment funded by companies is believed to be a central element in explaining economic competitiveness and specialization. These expenses are important for designing innovative technologies and, as a result, enhancing the innovation process.

Furthermore, these R&D investments have a significant effect on the economy’s cost competitiveness. G7 countries have changed their economies from imitating countries to a community of dynamic economies by concentrating on R&D spending and consistent policies. As a consequence, together with the digitalization of the economy. According to Trotta (2018) and Zhang et al. (2021), another significant determinant of promoting progress is the security of innovations and imagination. However, financial risk must be reduced in order to foster innovations by digitalization in the economy and business-financed R&D investment.

In some regions of China, a specific amount of shift in carbon emission is expected. Regardless of its potential to help policy formulation, the relationship and trans-impact between different regional emissions lack factual information (Tiep et al. 2021; Xia et al. 2020, and Asbahi et al. 2019). Evaluating countries’ performances instead of identifying common trends among several individual indicators are facilitated by composite indices (C.I.s) (Iram et al. 2020b; Baloch et al. 2020, and Chandio et al. 2020). The development of composite indicators is recorded through various literature.) established the DEA approach to build a CI, where they incorporated loss of information during the aggregation of different indicators through a non-compensatory MCDA approach (Liu et al. 2020a). The nexus between the paths of structural modernization and countries’ capacity is used by introducing the inclusive-sustainable transformation index. With its ability to protect the environment, the study focuses on a nation’s ability to developed a gender-inclusive services-based economy or modern industry (Khalfaoui et al. 2019; Anser et al. 2020c; Anser et al. 2020h, and Anser et al. 2020a).

The BRICS region countries are discussed by focusing on assessing their efficiency relative to technological innovation into sustainable development and transforming productive resources. Assessed energy efficiency for the BRICS region through the Super-SBM model. In addition to measuring the nexus between carbon emissions and energy efficiency, the bootstrap is used to modify values through the DEA approach, applied on small data samples. The efficiency of the relationship between CO_2_ emissions in the transport sector and the transport logistics’ performance measured through the Logistics Performance Index is analyzed by Yodkhum et al. (2017) and Acar (2018). Indicator selection is made through traditional techniques to aggregate indicators focusing on specific aspects. This process is not enough to provide general acceptance of the research and does not address each indicator’s weight. However, comprehensive index methods and relevant index sets to measure energy and environmental efficiency are missing in previous studies. With the help of a DEA-like composite index, this study analyzes the relationship between energy, financial, environmental sustainability, and social performance of BRIC and G7 countries. In addition to the possibility to have an outline of development at a regional level, the base of this study is formed over the reconstruction and modification of regional emissions and analyzing factors, such as energy, efficiency, and utilization. Most of the previous studies analyzed emission levels and variation based on actors related to energy efficiency, energy structure, financial development, production, industry, technological development openness, and population through some important approaches. The kaya components can decrease CO_2_ emissions through a model capable of improving energy efficiency and structure.

This study uses a DEA-like standard mathematical weight composite indicator to consolidate all the factors into a single measure. Multiple sets of indicators are used to develop composite indicators, which help measure energy financials and environmental efficiency. In order to develop an eco-friendly index for BRICS and G7 nations and calculate the mathematical aggregation, the study includes significant contributions conducted by past research.

The rest of the study is organized as follows: the “Methodology” section consists of the methodology; the “Results and discussions” section discusses the results and discussions, and the “Conclusion and policy implication” presents the conclusion and policy implications.

## Methodology

Table 1 represents a selection of the most recently developed relevant indicators based on multiple characteristics containing significant effects on country-wise policy measures, presenting a detailed instrument to investigate various countries’ tendencies. A set of indicator frameworks with new characteristics is included in this study (Wasif Rasheed and Anser 2017; Xu et al. 2020, and Ahmad et al. 2020). In short, there are two phases in the non-linear aggregation rule for building the climate change collaboration index. Build an outranking matrix by contrasting countries pairwise in terms of all sub-indicators. By utilizing the highest probability rating procedure, you will take account of any of the possible ranks. To extend the non-compensatory rule to the creation of a climate change collaboration map (Wang et al. 2019) suggest a heuristic rating method focused on minimum infringement ranking theory. The final results rating’s computing efficiency dependent on the non-compensatory law may be greatly increased using this technique.

**Table 1:** Indicators of sustainability performance

Environment	CO_2_ intensity	Energy use koe
	Carbon emissions per capita	Million ton
	CO_2_ from electricity generation	Million ton
	Ratio of forest area	% km^2^
	The proportion of public green space	%
	Emission of CH4	Mt CO_2_ equivalent
	SO_2_	
	PM2.5 air pollution	(% of total)
	NO_2_	
Energy	Energy consumption per capita	koe
	Carbon-based energy imports	Billion barrels
	Renewable energy sources	%
	Diversity in imported energy supply	%
	The time required to get electricity (days)	Days
	Access to electricity	(% of the population)
Financial	Energy intensity	MJ/$2011 PPP GDP
	R&D expenditure per capita	%
	Loan volume to GDP	%
	Arable Land	(% of land area)
	Capital	Current $
	GDP	billion$
	Merchandise trade	% of GDP
Social development	Life expectancy	Years
	Refugee Population	% population
	Contributing family workers	% of employment
	Employment in agriculture	% employment male
	Vulnerable employment	% employment male
	Death rate	Per 1000 People
	Literacy rate, adult male	% of male Ages

A DEA nonparametric frontier approach, proposed by Akpansung and Waziri (2018) and later expanded by, is used to establish the energy financials and environmental index. Many energy and environmental studies use the slack-based model (Anser et al. 2020h) and (Anser et al. 2020a). However, when companies are looking to minimize the undesirable output, desirable output maximization is utilized instead of maximizing model efficiency (Mohsin et al. 2020b; Mohsin et al. 2018b, and Mohsin et al. 2021). Contaminants, such as greenhouse gases and other contamination, are as much a part of the production process, making it essential to have environmental performance and financial growth in a well-maintained balance (Anser et al. 2020b), and (Anser et al. 2020f).

In this context, underlying energy, financial, and environmental indicators are included in this study. The multidimensional perspectives are assessed and summarized with the help of CI (Liu et al. 2020b; Lin et al. 2020; Jun et al. 2020, and Chien et al. 2021). Several evaluation systems are used for such fundamental indicators. At the national level, energy, financial, environmental, and carbon emissions are measured with C.I.s, used to test, evaluate, and propose policy (Li et al. 2018; Sun et al. 2019a). Different features with substantial influence on policy changes from the base for different features used in decision-makers. Financial, energy, and environmental indicators are combined with the performance assessment of BRICS. Let the number of components for energy vector, financial, and environmental variable be *n*. Then the following should represent the model:

1 $$ {\displaystyle \begin{array}{l}{\mathrm{gI}}_i=\max \sum \limits_{j=1}^n{W}_{ij}^g{I}_{ij}\\ {}\end{array}} $$

$$ {\displaystyle \begin{array}{c}\mathrm{s}.\mathrm{t}\kern0.5em \mathrm{g}{\mathrm{I}}_i\ {\sum}_{j=1}^n{W}_{ij}^g{I}_{kj}\le 1,k=1,2,\dots, m\\ {}{W}_{ij}^g\ge 0,j=1,2,\dots, n\end{array}} $$

Now

2 $$ {\mathrm{bI}}_i=\min \sum \limits_{j=1}^n{W}_{ij}^b{I}_{ij} $$

$$ {\displaystyle \begin{array}{c}\mathrm{s}.\mathrm{t}\kern0.5em \mathrm{b}{\mathrm{I}}_i{\sum}_{j=1}^n{W}_{ij}^b{I}_{kj}\le 1,k=1,2,\dots, m\\ {}\kern0em {W}_{ij}^g\ge 0,\kern0.5em j=1,2,\dots, n\end{array}} $$

The objective function of the proposed model is to combine these 2 models (Mohsin et al. 2019b), (Mohsin et al. 2020a; Mohsin et al. 2021). If tie-breaking is needed, remove the corresponding countries where ties exist to establish a new sub-outranking matrix, re-rank the subset of countries using the steps above, and then combine with previous ranking results to obtain a full ranking. Suppose the summations of all countries in the sub-matrix are still equivalent in the second stage. In that case, a country can be selected at random as the subset’s representative, and the procedure can be replicated to achieve a final full order. The definition of strength degree can be translated as the values of each factor (for example) in the outranking matrix, which supports the following statement: In the very least, the nation succeeds admirably. The higher the importance, the more effective the strength. Consequently, the sum of the row vectors in the outranking matrix can be used to determine a country’s total intensity degree in contrast to all others. As a consequence, it seems fair to offer a nation a higher ranking when it achieves a higher standard. In contrast to the original procedure’s complexity, the heuristic procedure will obtain the final full rating of countries’ cooperation success by measuring only intensity degree ratings. The number of scores that must be measured is largely decided by the probability of future relations between countries:

3 $$ \left(\mathrm{CI}\right)\lambda =\lambda \frac{gI_i- gI}{gI^{\ast }- gI}+\left(1-\lambda \right)\frac{bI_i- bI}{bI^{\ast }- bI} $$

The constructed index is compensation-proof due to the non-linear aggregation theorem and is thus named for the non-compensatory index (Tyagi et al. 2020; Çelik et al. 2018). In comparison, the non-compensatory index includes a variety of attractive features. Trade-offs between sub-indicators are not tolerated owing to the non-compensatory characteristic. Consequently, the sub-indicators weight may be stated as the magnitude of a significant coefficient. Non-compensatory will, therefore, prevent the problem of double weighting. It also satisfies neutrality, Pareto optimality, monotonicity, and, most significantly, reinforcing. The definitions of Pareto optimality and monotonicity are clear. According to neutrality, the global change partnership index resulting from the non-compensatory law would handle both countries fairly. Suppose a country gets the same rating from the corresponding subset of sub-indicators. In that case, reinforcement means getting a consistent ranking outcome in terms of a mixture of the various subsets of sub-indicators. When dealing with several hierarchical indicator structures, this property comes in handy (Becker et al. 2017; Gygli et al. 2019).

Despite its merits, the non-compensatory index has certain shortcomings that must be recognized by utilizing it to measure climate change cooperation results. Since it is determined by pairwise contrast, the outranking matrix only includes sub-indicators’ ordinal details, meaning that the cardinal information underlying individual sub-indicators is almost entirely overlooked. As a consequence, we cannot use the non-compensatory rule’s findings to evaluate the difference in cooperation success between two countries. Furthermore, as Nasir et al. (2019) point out, as the number of countries rises, the final rating computation will rapidly become unmanageable. The output of a total of G7 countries will be reassessed in this report, and the resulting permutation will be. Among the shortcomings, the sophistication of the computation could be the most important issue that prohibits the non-compensatory rule from being applicable to the assessment of climate change cooperation results.

Calculate the summations of row vectors in the outranking matrix to decide which countries are listed first. A country with a higher summation earns a higher score. The process comes to an end if there are no relations. The climate change collaboration index will now be achieved using the outranking matrix and the heuristic ranking method in either scenario. However, Diesendorf and Wiedmann (2020) reported that indifferent local relation might be global indifference due to the elements’ transitivity property inside the outranking matrix. The definition of indicator thresholds is introduced into the construction of the outranking matrix to increase robustness. If the total gap between two countries’ respective indicator output is smaller than or equivalent to an indifference threshold, their performance on the sub-indicator is indifferent.

Similarly, the preference relationship between the output of two countries under the sub-indicator should satisfy and, where is the corresponding preference threshold if a new binary relationship between countries arises, namely, poor choice. The current partnership converts Eq. (1) into the following equation, which can create the outranking matrix.

### Econometric specification

To measure various financial estimations, econometrics is one of the most accepted techniques (Hansen 1982). Econometric does not require a comprehensive understanding of data allocation necessary for the maximum likelihood estimation (MLE). However, a specified moment generated with the original model is required for this method (Asif et al. 2020; Sarker et al. 2020; Iram et al. 2020a, and Tehreem et al. 2020). The computation of econometric is easy for available data allocation, unlike the computation of MLE. Model for log-normal stochastic volatility can be considered as an excellent example of this process. The econometric estimation approach’s ability to present a definite direction for the models bounded by multiple moment conditions and moments model parameters makes this approach unique (Poudineh et al. 2020; Topcu and Payne 2018). It becomes difficult to apply the traditional static estimation methods due to the dynamic panel data model’s endogenous problems (Anser et al. 2020e; Steffen 2018, and Anser et al. 2020d).

The standard linear regression model is shown in equation (4). The lag terms used in this process are to be represented as:

4 $$ {\mathrm{GEPI}}_{it}=\upalpha +{\upbeta}_1\ {\varphi}_1+{\upbeta}_2\ {\varphi}_2+{\upbeta}_3\ {\varphi}_3+{\upbeta}_4\ {\varphi}_4+\upbeta {\mathrm{GEPI}}_{i,t-1}+\upgamma \mathrm{P}.\mathrm{S}{.}_{it}+\uptheta {X}_{it}+{u}_t+{v}_i+{\varepsilon}_{it} $$

where β1, β2, and β3 are unknown coefficients, and α represents the intercept. The energy indicator is given as β_1_ *φ*_1_, the financial indicator as β_2_ *φ*_2_, the environmental indicator as β_3_ *φ*_3_, and indicators from social development as β_4_ *φ*_4_. Since the current green performance growth index is hugely affected by the green performance index, the control variables set is signified as *X*_it_, fixed time effect as *u*_*t*_, single fixed-effect as *v*_*i*_, and random error term as *ε*_it_ (Yousaf et al. 2020; Tehreem et al. 2020; Wasif Rasheed and Anser 2017, and Xu et al. 2020). The set of each indicator in comparison with each dimension is shown in Table 1 However, the differencing method is likely to cause some problems, starting with eliminating the individual effects (Ikram et al. 2019a; Shah et al. 2019). A negative relationship between the first differences and one-step system econometric is suggested through the two system econometric. It is possible to trigger higher efficiency through an econometric estimator, as it initiates more instruments (Ikram et al. 2019a; Sun et al. 2019b, and Ikram et al. 2019b).

## Results and discussions

This section presents the results and discussions using the proposed methodology. Per capita energy usage of China and Russia is higher than the other countries in the zone, as shown in Table 2.

**Table 2:** Energy and environmental efficiency

Countries	Brazil	Russia	India	China	South Africa
Energy Eff	0.78	0.91	0.88	1	0.82
Env Eff	0.80	0.90	0.82	0.98	0.94

The accumulative average score for the environment efficiency is recorded at 0.92 to 0.98, whereas in 2016, the environmental efficiency score for China and South Africa is recorded at 0.98 and 0.92. 0.97 to 0.94 is the recorded range for energy efficiency, with India recorded as the maximum and South Africa as the minimum. A mere 0.9% increase in emissions of CO_2_ is recorded through the results. As the biggest threat to environmental conservation, carbon emission in Russia declined by 1.5%, whereas a 7.8% and 3.3% rise is recorded for India and Brazil, respectively. The per capita energy consumption for the various countries is shown in Table 3.

**Table 3:** Per capita energy usage (koe)

Country	Brazil	Russia	India	China	South Africa
Energy use (koe)	1496	4943	637	2237	2695

Canada’s ability to cope with energy, financial development, and environmental sustainability is evident through the overall composite index scores, with Canada recorded at 0.72. Considering factors, such as self-energy resources, higher financial production, and low carbon pollution, Canada shows better performance than other countries. Recorded at 0.72 and 0.62, France and Italy have secured second place, regardless of the insufficient energy resources, followed by Germany and Japan recorded at 0.63 and 0.50, respectively. The USA’s average efficiency score was recorded at the lowest value of 0.40, regardless of the high financial development. In order to stimulate green consumption and build an ecological civilization, people are motivated to submit environmentally friendly materials amid the fast-renewable energy development seen in many regions, and a −1.7% decrease in emissions is evident through the changes in energy structure. The southwest region with rich gas emissions has a good emission reduction performance (−0.5%), which is better in the southwest region. The comparison with BRICS shows that the BRICS’s overall environmental efficiency index score in Table 4 shows the CO_2_ emission per capita for the BRICS region.

**Table 4:** CO_2_ emission per capita (metric tons)

Country	Brazil	Russia	India	China	South Africa
CO_2_ emission per capita	2.39	11.51	1.1	4.26	6.95

Digitalization of the economy is important for a country’s economic development, and technical progress is unlikely without it. It also has a huge effect on factor productivity. The G7 countries’ impressive achievement in maintaining strong economic growth is attributed to accelerated technical progress and other factors. As a consequence of developments in information and communication technology (ICTs), the digitalization of markets has altered the market dynamics of G7 countries in recent years. In terms of improved growth, societal transformation, and industrial progress, these digitalization developments have helped the G7 economies. These seven countries manage 58% of the world’s net assets.

Furthermore, the G7 countries’ economic output has been slowly improving over time. Furthermore, these great seven economies invest billions in research and development (R&D), improving their economic and innovation success. As a consequence, it is important to explore the effect of digitalization and R&D on technical progress in the G7 countries. As a consequence, the G7 countries hold a substantial portion of the world’s net income, have accelerated their technical growth, and have made strides in digitalization. The literature on the variables that affect technological advancement is comprehensive. Wage, imports, human resources, operational efficiency, financial growth, debt servicing, corruption, knowledge spillovers, and, most significantly, R&D spending are all variables that affect technical advancement.

The regional development in the two regions has been significantly affected by the financial development rates of BRICS and China. A reduction recorded at −2.7%, −1.3%, −0.8%, and −0.4% of CO_2_ emissions is evident in the Northwest, Northeast, North China, and Central regions, respectively, due to the relatively weak financial growth and high energy consumption.

**Fig. 1:**
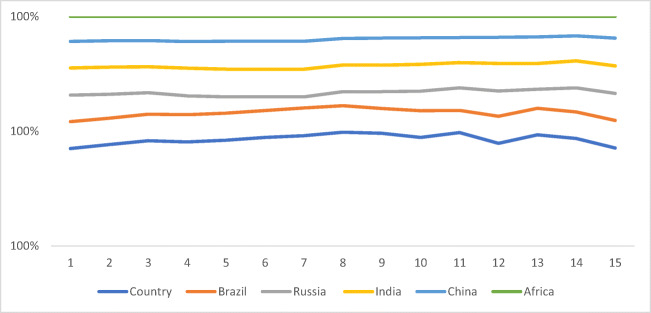
Overall environmental efficiency index score of BRICS

Figure 1 shows the overall environmental efficiency index score. The Human Development Index (HDI) for Germany, the USA, Canada, and Italy were recorded in the decreasing order as 0.916, 0.915, 0.913, and 0.873. The capability to assure energy security in supply, demand, and the production of green and other low-carbon energy supplies defines environmental sustainability. A range of 267.19 to 4.64 reflects the primary energy supply. A value of 482.79 is depicted by Canada, securing first place, with Germany at second position with a recorded score of 34.87, whereas Table 5 shows the minimum value for Japan, recorded at 4.64.

**Table 5:** Overall Environmental Efficiency Index Score of BRICS

Country	Brazil	Russia	India	China	South Africa
2002	0.94	0.93	0.95	0.93	0.87
2003	0.92	0.84	0.95	0.92	0.85
2004	0.93	0.75	0.91	0.92	0.84
2005	0.95	0.66	0.97	0.93	0.88
2006	0.95	0.57	0.97	0.98	0.87
2007	0.94	0.48	0.97	0.98	0.87
2008	0.97	0.39	0.97	0.98	0.87
2009	0.93	0.49	0.94	0.93	0.77
2010	0.87	0.59	0.93	0.95	0.76
2011	0.94	0.69	0.94	0.93	0.75
2012	0.78	0.79	0.89	0.88	0.74
2013	0.95	0.89	0.97	0.92	0.73
2014	0.93	0.67	0.91	0.93	0.72
2015	0.93	0.85	0.95	0.88	0.68
2016	0.97	0.95	0.97	0.98	0.76

A variation between 22.03 and 6.31% insinuates the importance of renewable energy production. The highest value of 22.03% is recorded for Canada, with Italy and Germany at 16.52% and 14.21%, whereas Japan possesses the maximum value, recorded at 6.31%. An extensive, energy-intensive usage is evident as a result of increased energy sources in Germany and France. The overall environmental efficiency index score of BRICS. Considering CO_2_ emission efficiency, France and Italy proved to be equally competent and well-performed. In contrast, the least competent countries include Japan, Canada, and the USA among all G7 members for more or less every year. A significant variation can be seen in the emission efficiency of CO_2_ among G7 economies, with a collective score of less than 0.50. According to the study, the environmental condition can be improved with the help of energy consumption efficiency and emission efficiency of CO_2_, concluded through an analysis of the relationship between the two factors. Following a value of 70.3%, 82.4%, and 91.8% in 2001, the value between the frontier and BAU dropped to 45.1%, 30.7%, and 54.2% in 2015, pertaining to the widening difference between business as usual (BAU) and the frontier CO_2_ intensity in the petroleum, electricity, and chemical sectors. With small fluctuations, a rather stable value of CO_2_ intensity difference between frontier and BAU is evident for the ferrous metal, whereas an increase of 23.1% and 76.5% in 2015 from 7.5% and 69.4% in 2001 was seen between the BAU and frontier of non-ferrous metals and non-metallic products. The energy efficiency of G7 economies is shown in Fig. 3, where all types of equity and environmental sustainability were assessed to achieve the given results. The UK and the USA are the lowest performing countries according to the energy intensity and environmental index analysis. Without affecting energy consumption (i.e., compromising emission levels), maintaining financial development is highly challenging for a country. Ye et al. established how energy and environmental efficiency are primarily impacted by energy consumption and financial growth.

**Fig. 2:**
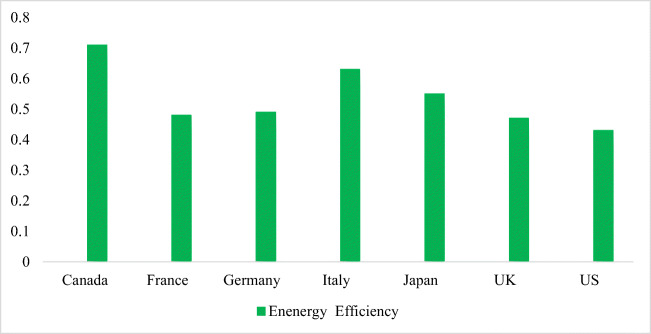
Energy efficiency

Figure 2 shows the energy efficiency score. Hence, it is vital to consider the direct impact of energy consumption on developing financial factors when formulating energy management policies. It is possible to aid energy security and reduce CO_2_ emissions if meaningful measures are established to improve energy efficiency. Consequently, the formulation of a policy framework should consider energy, financial, and environmental efficiency. Moreover, countries should be assessed based on their ability to save energy and reduce carbon emission. The environment is affected locally, regionally, and globally by increasing energy consumption and energy efficiency through increased efforts to generate clean energy. Therefore, environmental and financial factors should be considered for energy efficiency estimates.

As compared to the other G7 nations, Italy has improved its energy efficiency score due to the high-energy consumption rates. For carbon dioxide emissions and the overall performance, indicators, such as carbon intensity (carbon/GDP), total energy intensity (energy/GDP), per capita carbon dioxide emissions, and carbon factors (carbon/energy), are considered to assess the performance of various countries. Energy, financial and environmental efficiency, and CO_2_ emissions have been measured in this study to help factors such as energy, economy, and various environmental indicators. With the financial efficiency score as an output, the environmental efficiency is measured by fixing the best undesirable output, according to the financial and environmental efficiency scores of the G7 countries. With scores below 1, France and Canada are the least efficient countries. OECD member nations consume 55 btoe of energy, with a real gross domestic product considering of $39 trillion, showing the similar state of G7 countries and the OECD region in terms of energy consumption, GDP, and CO_2_ emission, which constitutes 75% of the world’s real GDP and 42.0% of the world’s total energy. An estimate of bmt 13 CO_2_ release was estimated for the primary energy extracted from oil and coal in 2010 (Sun et al. 2020c, Sun et al. 2020a. and Sun et al. 2020b).

China’s advanced economy requires 18.0% of global energy consumption. With a decrease in percentage over time, oil was retained as the core energy source with a 33.0% share. Significant global political pressure has been enforced on G7 and OECD countries to decrease their emissions. Resulting in a firm cut down to a value equivalent to that of the year 2000, 4.7% utilization of energy by OECD nations was recorded in 2009. With an assurance of preserving the environment for both current and future generations, it is vital to execute sustainable development. The higher GHG emissions are mainly due to the countries consuming renewable energy in low quantities (Australia, Belgium, the Netherlands, and Israel) in their nationwide demand for energy, supporting renewable energy argument resulting in decreased CO_2_ emission levels. Therefore, renewable utilization is low for countries with higher emissions (Alemzero et al. 2020b), (Sun et al. 2020a) and (Alemzero et al. 2020a).

**Fig. 3:**
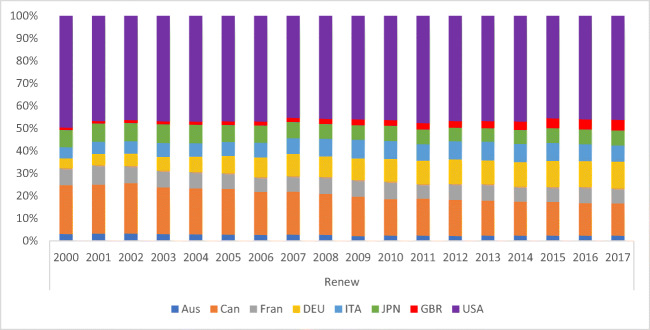
Renewable energy of G7 countries

The political economy mostly provides the financial base for assessing the extent of energy efficiency against per unit of power output (Fig. 3). With a specialized focus on analyzing total-factor efficiency, studies such as China and Germany have contributed to energy efficiency. A study supports the findings of this study. Compound energy inputs, different materials, and resources, such as greenhouse emissions, have been focused on by the combined production method used in these researches and calculated the rank-based assessment of electricity regarding this process. Analyzing a total-factor structure through the DEA analyzed the energy efficiency in the G7 (Japan, Canada, the UK, France, Italy, and Germany). The development of the financial model requires factors that carry out measures with higher energy performance. Focusing exclusively on the investigation of energy potency and the environmental Kuznets curve, a replacement information-intromission analysis technique was considered to provide environmental upgrading submissions.

### Energy efficiency vs. environmental index

In the transportation sector, petroleum products are responsible for more than 90% of energy consumption. CO_2_ emission is continuously increased due to an increase in transportation demand, consuming more natural gas. With a one-way causal relationship between GDP growth and pollutant emissions for the G7 countries, declining energy efficiency is dependent on several strategies, policies, and technologies, related to the overall energy consumption for the G7 countries. Energy efficiency improvement has caused a decrease in the G7 economies’ energy intensity during the year. The energy efficiency (E.E.) and environmental index (EIN) for the G7 countries are shown in Table 6.

**Table 6:** Energy efficiency and environmental index

Country	EE	EIN
Canada	0.71	0.62
France	0.48	0.51
Germany	0.49	0.46
Italy	0.63	0.68
Japan	0.55	0.53
UK	0.47	0.76
USA	0.43	0.79

**Fig. 4:**
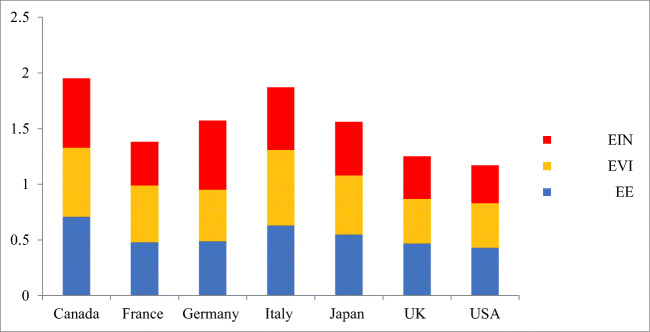
Trend of E.E., EVI, and EIN

**Fig. 5:**
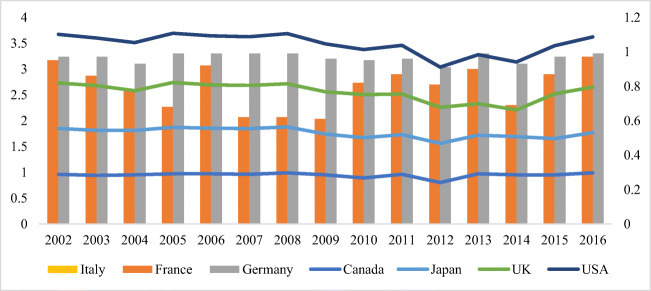
Overall efficiency score of G7

A total decoupling in stable energy utilization was observed in Italy, Japan, the UK, and the USA. Eco-friendly pressure from fossils-based energy utilization and production decreases due to the absolute decoupling. The mixture of solar, hydro, wind, and additional energy resources constituting independent energy production could be the reason. The empirical evidence of the given process is far from the scope of this study. Environmental deterioration and climate vulnerability are decreased by renewable energy. The index of energy efficiency trend, environmental index, and energy intensity index is given in Fig. 4. In addition to the concrete and effective policies, Canada also has energy security and vast crude oil reserves. The efficiency score of G7 countries is shown in Fig. 5.

### Econometric analysis

The results of estimates calculated with different estimation tools are given in Table 7. The effect of fiscal spending for R&D on GEPI is shown in column 1 and 2 of the table, whereas the impact of public spending in terms of education on GEPI is observed in columns 3 and column 4. Both column 1 and column 3 help in observing the result of the one-step system econometric model. The one-step system econometric hypothesis consists of biased estimates considered by this study for comparison. Sargan test and the null hypothesis of second-order auto-correction, fixed through the AR (2) test, were used to expose the instrumental validity’s credibility. The econometric estimation in this study is validated with the help of the two given tests.

**Table 7:** the impact of energy, financial, environmental, and social development

	Energy and environment						Financial and social development					
Classes	The specific impact of energy and environmental			The specific impact of energy and environmental			The general effect of financial and social development			The general effect of financial and social development		
				Demand-pull	Demand push	Balanced				Demand-pull	Demand push	Balanced
Dependent variables	ln(E. C._F, t_)		E. C._f, t_	ln(E. C._f, t_)			ln(ECO_f, t_)		ECO_f, t_	ln(ECO_f, t_)		
Methods	i.	ii.	iii.	iv	v	vi	vii	viii	ix	x	xi	xii
Mix of policies	-0.0865***	-0.0832***	-1.399***	-0.0542	-0.0874***	-0.410***	0.131***	0.126***	0.392***	0.199***	0.101***	0.121
	(0.0321)	(0.0256)	(0.312)	(0.0626)	(0.0317)	(0.112)	(0.0326)	(0.0188)	(0.0823)	(0.0519)	(0.0352)	(0.129)
Constant	Yes	Yes	Yes	Yes	Yes		Yeses	Yes	Yes	Yes	Yes	Yes
*φ*_1_	Yes			Yes	Yes		Yes	Yes	Yes	Yes	Yes	
*φ*_2_	Yes	Yes		Yes		Yes	Yes			Yes	Yes	Yes
*φ*_3_	Yes			Yes		Yes	Yes	Yes		Yes	Yes	Yes
*φ*_4_	Yes			Yes	Yes			Yes				Yes
Supporting Volunteer CSR	Yes	Yeses	Yes	Yes	Yes			Yes				Yes
*R*^2^	0.019	0.023		0.015	0.023	0.014	0.042	0.042		0.032	0.042	0.031
Observations (N)	59,039	59,042	60,065	60,748	62,400	48496	58,521	65,521	64,521	54,188	59,212	50,769

^a^*, **, and *** Specify statistical importance at the 10 %, 5%, and1% Significance a respectively

^b^The inflation factor is modified according to all the dependent variables based on 1998

^c^Models (3) and (9) are calculated using generalized linear models (GLM) with gamma distribution and log relation functionality.

Infrastructure and R&D expenditure are all affected by national revenue. As a result, a rise in a country’s income boosts the G7 countries’ innovation. Fourth, the results indicate that the financial danger index has a detrimental effect on creativity, confirming the findings of International Energy Agency (2019). High financial risk contributes to low projected returns, which decreases the supply of bank funding for R&D. Because of the high financial danger that companies face, banks are also slow to support new concepts. As a consequence, high financial risk is seen as a threat to technical progress in the G7 countries. G7 countries must minimize financial risk to promote technology through digitalization in the economy and business-financed R&D investment. To conclude, the economy’s digitalization and R&D investment have important consequences for technical advancement. However, in order to enjoy the rewards of these trends, the G7 countries must reduce their financial risk.

This analysis focuses on the G7 nations, which are composed of seven main developed economies. However, the G7’s exposure to global economic growth is fluctuating, with the share of growth dropping from 24.1% in the 2000–2005 era to only 9% in the 2005–2010 period. The global financial crisis of 2007–2008, which triggered major losses to the G7 economies, was one of the key explanations for this substantial reduction in global development contribution. Nonetheless, between 2010 and 2017, these markets jointly recovered and accounted for 17.1% of global expansion. The G7 countries were selected mainly because of the “institutional collapse theory,” which ties a country’s natural resource curse to its low institutional consistency, which is mostly valid for developing countries. The G7, on the other side, are developing nations, and likely their administrative quality and, as a result, effective resource utilization can turn a curse into a blessing. These results are supported by the outcomes of alternative requirements in the table. Several essential findings are highlighted by Mohsin et al. (2018b), Mohsin et al. (2019b), Sadiq et al. (2020), and Chien et al. (2021), along with regression.

**Fig. 6:**
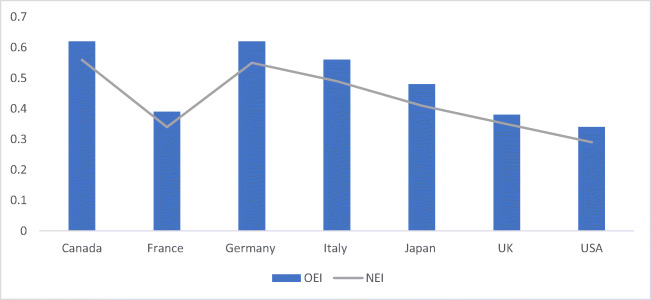
Original and newly simulated values

The efficiency index for energy and environment through DEA-Like composite indicator and econometric based estimation has been generated. It is in line with the sensitivity assessment of data uncertainty for robust econometric is consequently carried out, and for data errors, an assumption within ±20% is used. The range of [−20%, 20%] using random numbers has been produced to obtain the energy and environmental index. Following the uncertainty used to consider the data accuracy, the values fluctuate significantly, providing a comparison between the value of coefficients developed with the original dataset’s help and the newly developed dataset meant for sensitivity analysis. Figure. 6 presents some minor changes with the change of data. Table.8 presents the robustness analysis.

**Table 8:** Robustness estimation for one-step and two-step econometric

	One step	Two-step	One step	Two-step
	(1)	(2)	(3)	(4)
L.GEPI	−0.221***	−0.089**	−0.221***	−0.079**
	(0.041)	(0.039)	(0.037)	(0.043)
PCRD	0.057**	0.044***		
	(0.08)	(0.021)		
PCEDU			0.0492	0.077***
			(0.073)	(0.031)
INDUS	−0.413***	−0.463***	−0.383***	−0.386***
	(0.073)	(0.067)	(0.083)	(0.07)
Green	0.083**	0.078	0.088**	0.067*
	(0.045)	(0.043)	(0.042)	(0.042)
GDPLA	0.085***	0.041**	0.095***	0.036*
	(0.05)	(0.021)	(0.076)	(0.028)
Openness	0.021	−0.028*	0.016	0.012
	(0.022)	(0.022)	(0.022)	(0.032)
Constant	3.034***	3.384***	3.264***	3.374***
	(0.54)	(0.472)	(0.733)	(0.382)
Observations	302	302	302	302
Adjusted *R*^2^	0.086		0.085	
Arellano-bond AR (1)		−6.732		−6.463
		[0.000]		[0.000]
Arellano-bond AR (2)		−0.567		−0.762
		[0.739]		[0.713]
Sargan test		271.231		281.658
		[0.702]		[0.723]

*p*-value in brackets and standard errors in parentheses

*p<0.1

**p<0.5

***p<0.01

Although a favorable relationship between natural resources and financial growth could be a factor in G7 countries’ superior institutional consistency and productivity, this hypothesis will not be tested in this analysis. In addition, potential studies may involve variables such as inflation, financial globalization, technical progress, and energy use. This result can be related to the key factors of inequalities and CO_2_ pollution at the moment. Inequality plummeted between 1950 and 1975 due to the new agreement in the United States, the emergence of Keynesianism in Western Europe, egalitarian taxes, and the gradual growth of social welfare systems. The relationship between income disparity and CO2 emissions between 1950 and 2000 indicates that income redistribution and gross CO_2_ emissions are mutually exclusive. As it is known in the literature, the equity pollution problem is the concept that income redistribution may increase aggregate pollution. The equity pollution problem could have consequences for redistribution policies. On the surface, it seems that wealth redistribution programs, in the absence of policies to encourage clean energy sources, would lead to greenhouse warming. However, as Sokołowski (2019) points out, the equity pollution problem does not inherently suggest that income redistribution is undesirable; however, optimal redistribution would entail comprehensive welfare research and a variety of hypotheses regarding household welfare, market dynamics, and acceptable social outcomes.

## Conclusion and policy implications

This work analyzes the relationship between energy, financial, and environmental sustainability and social performance of BRICS and G7 countries in the period 2000–2017 through a DEA-like composite index. A holistic combination approach was adopted for the multiple indicators sets to mitigate an information loss criterion of the composite index during the aggregation process. Each nation was analyzed through the energy, financial, environmental, and social performance ratings and specific metrics to obtain additional information on each country’s performance from suitable indicators. The significant findings of the study are listed below.

The multiple indicator analysis shows Canada’s dominance over other countries. These indicators include energy self-sufficiency ratio, energy dependence, and per capita energy consumption. Fossil fuel consumption is a major contributor to global warming; fossil fuel-based energy generation must be replaced by renewable, sustainable energy generation. A 0.18%, 0.27%, 0.22%, 0.09%, 0.31%, and 0.32% reduction in carbon emission is observed with a 1% increase in R&D costs by Canada, France, Germany, Italy, Japan, and the USA, respectively. In all nations, except Japan, the turning point for sustainable environmental conditions was between $6933 and $36,255.

Among the G7 countries, the USA is vulnerable with respect to the energy, financial, social, and environmental indicators. Canada has the best financial, environmental, and social performance score for oil, which translates into a greater capacity for it than the other G7 nations to preserve its self-sufficiency in energy, financial growth, and environmental sustainability. France, Italy, and Japan follow, respectively, while the USA ranks at the bottom in energy, socio-financial, and environmental performance with a score of 0.40. Despite the G7 countries’ developed economies, their environmentally fragile condition shows the overall global status in terms of environmental sustainability. In this context, the following policy changes are proposed for policymakers and government officials.

Corporations should adopt environmental safety policies to promote zero-emission awareness by providing green parks and developing green footprint. The state should also focus on reducing pollutant emissions and the conservation of electricity under the Paris Agreement by supporting and implementing energy efficiency strategies. Governments should regulate rising energy demand because the stakeholders should endorse contemporary and increasing concerns about sustainable development and global warming globally. Strong policies and processes to increase the supply of sufficient clean and affordable energy should be placed in place to sustain a stable environment without restricting financial growth and development. Based on these observations, the following suggestions can be made: First, it seems that the G7 countries have effectively escaped the resource curse pit. That may be because of their agencies’ efficiency, which policy capacities can assess in oversight and evaluation, public investment management, and budget procedures. As a consequence, politicians must be mindful of this aspect and adapt it to the productive usage of natural capital. Second, when designing strategies to strengthen financial growth metrics, the G7 economies must emphasize oil market stability. To promote faster long-term financial growth, policymakers must put an end to or at least restrict energy price fluctuations. This proposal, though, would be unnecessary unless adequate frameworks for achieving this policy objective were enforced. Finally, policymakers can ensure that the stock market is open and that it offers opportunities for prospective buyers and creditors to link, which would help to expand capital formation and, in turn, boost financial growth.

## Data Availability

The data that support the findings of this study are attached.

## Abbreviations

BA: Business as usual
CI: Composite index
DEA: Data envelopment analysis
GDP: Gross domestic product
GHG: Greenhouse gases
HDI: Human Development Index
MCDA: Multi-criteria decision analysis
OECD: Organization for Financial Co-operation and Development
R&D: Research and development
SDG: Sustainable Development Goals
